# Low MicroRNA-19b Expression Shows a Promising Clinical Impact in Locally Advanced Rectal Cancer

**DOI:** 10.3390/cancers13061456

**Published:** 2021-03-22

**Authors:** Jaime Rubio, Ion Cristóbal, Andrea Santos, Cristina Caramés, Melani Luque, Marta Sanz-Alvarez, Sandra Zazo, Juan Madoz-Gúrpide, Federico Rojo, Jesús García-Foncillas

**Affiliations:** 1 Cancer Unit for Research on Novel Therapeutic Targets, Oncohealth Institute, IIS- Fundación Jiménez Díaz-UAM, E-28040 Madrid, Spain; jaime.rubiop@quironsalud.es (J.R.); andrea.santos@quironsalud.es (A.S.); ccarames@fjd.es (C.C.); 2 Translational Oncology Division, Oncohealth Institute, IIS- Fundación Jiménez Díaz-UAM, E-28040 Madrid, Spain; 3 Medical Oncology Department, University Hospital “Fundación Jiménez Díaz”, UAM, E-28040 Madrid, Spain; 4 Pathology Department, IIS- Fundación Jiménez Díaz-UAM, E-28040 Madrid, Spain; melani.luque@quironsalud.es (M.L.); marta.sanza@quironsalud.es (M.S.-A.); szazo@fjd.es (S.Z.); jmadoz@fjd.es (J.M.-G.); frojo@fjd.es (F.R.)

**Keywords:** MiR-19b, locally advanced rectal cancer, prognosis, pathological response

## Abstract

**Simple Summary:**

The establishment of molecular markers to predict response to neoadjuvant chemoradiotherapy (CRT) would help to avoid unnecessary toxicities and surgery delays in the clinical management of locally advanced rectal cancer (LARC) patients. Our aim here was to in-vestigate the clinical impact of miR-19b in this disease. Interestingly, our findings highlight the potential usefulness of miR-19b as a predictor of response to neoadjuvant CRT and outcome, and suggest PPP2R5E as a relevant miR-19b target in LARC.

**Abstract:**

The standard treatment for patients with locally advanced colorectal cancer (LARC) is neoadjuvant 5-fluorouracil (5-FU) based chemoradiotherapy (CRT) followed by surgical mesorectal excision. However, the lack of response to this preoperative treatment strongly compromises patient outcomes and leads to surgical delays and undesired toxicities in those non-responder cases. Thus, the identification of effective and robust biomarkers to predict response to preoperative CRT represents an urgent need in the current clinical management of LARC. The oncomiR microRNA-19b (miR-19b) has been reported to functionally play oncogenic roles in colorectal cancer (CRC) cells as well as regulate 5-FU sensitivity and determine outcome in CRC patients. However, its clinical impact in LARC has not been previously investigated. Here, we show that miR-19b deregulation is a common event in this disease, and its decreased expression significantly associates with lower tumor size after CRT (*p* = 0.003), early pathological stage (*p* = 0.003), and absence of recurrence (*p* = 0.001) in LARC patients. Interestingly, low miR-19b expression shows a predictive value of better response to neoajuvant CRT (*p* < 0.001), and the subgroup of LARC patients with low miR-19b levels have a markedly longer overall (*p* = 0.003) and event-free survival (*p* = 0.023). Finally, multivariate analyses determined that miR-19b independently predicts both patient outcome and response to preoperative CRT, highlighting its potential clinical usefulness in the management of LARC patients.

## 1. Introduction

Colorectal carcinoma (CRC) is a highly prevalent multifactorial disease in the Western world, being the third leading cause of death from cancer. In Spain, more than 44,000 new colorectal cases are diagnosed each year and, unfortunately, around 15,000 deaths are also reported annually [1]. Rectal carcinoma represents almost 30% of all diagnosed colorectal cancers. The rectum is by definition the continuation of the sigmoid colon, measuring between 12 and 15 cm and continuing up to the dentate line or anal verge [2]. Although new therapeutic approaches are currently under study, the standard treatment recommended by European Society for Medical Oncology (ESMO) guidelines for locally advanced rectal cancer (LARC) is a preoperative chemoradiotherapy (CRT) based on 5-fluorouracil (intravenous or oral formulation, capecitabine) or short-course preoperative radiotherapy (SCPRT) followed by a total mesorectal excision (TME) [3]. Of note, several studies have demonstrated that this therapeutic strategy leads to a better local response and a lower local relapse rate compared to TME alone or followed by adjuvant CRT [4,5]. With this management, around 20% of pathological complete responses (pCR) are reached, and these patients are the ones who benefit most, with better overall survival (OS) and disease-free survival (DFS) [6,7]. Those patients who do not achieve a pCR are those with a higher probability of local and distant relapse, with a 30% of cases developing recurrences within the next 10 years from initial treatment. Despite significant improvements in clinical management in the last decade, distant recurrences remain the major cause of mortality in these patients [8]. In fact, it would be very valuable to identify effective markers with predictive value for disease prognosis or recurrence. Then, clinicians could manage differently those subgroups of patients candidate to be resistant to chemoradiotherapy or those that likely reach a pCR before treatment in order to reduce undesirable observed morbidities and mortalities as well as delays in the resection of the primary tumor [9]. Although researchers have identified several postsurgical prognostic markers as well as few preoperative markers, none of them have been established clinically to date [10,11,12]. Thus, a major current limitation for clinical management of LARC patients is the absence of effective predictors of pathological tumor response before neoadjuvant CRT.

MicroRNAs (miRs) are small single-stranded RNA molecules (between 19 and 25 nucleotides) that do not code for proteins but regulate the expression of other genes at the post-transcriptional level. They bind to specific sequences of 3’ Untranslated Region (UTR) regions of the target messenger RNA (mRNA) and impair their translation [13]. In this way, miRs act as oncogenes or as tumor-suppressor genes, depending on the gene to which the translation is repressed. The clinical value of miRs in human cancer is well known and has been largely reported in many tumor types including rectal cancer, and they can be used to improve tumor diagnosis, as prognostic markers or as predictive markers of response to treatment [14,15,16]. They can also be used to predict the tumor resistance to therapeutic agents [17]. In rectal cancer, miRs have shown to be potential useful biomarkers based on their detection in both tumor tissue and liquid biopsies [12]. Consequently, the assessment of levels of a single miR or miRNAs-based signatures in tumor specimens or circulating miRs/tumor cells in body fluids could improve the classification of LARC patients according to the CRT response, thus facilitating the clinical decision [18,19,20,21]. MiR-19b has been widely associated with carcinogenesis in various tumor subtypes through different signaling pathways. MiR-19b overexpression has been reported to promote tumor growth and metastasis through targeting p53, which led to decreased BCL2 Associated X, Apoptosis Regulator (BAX) and p21 levels [22]. MiR-19b has also been described as an inhibitor of Phosphatase And Tensin Homolog (PTEN) and activator of the Phosphoinositide-3-kinase (PI3K)/Protein Kinase B (AKT) signaling in Wilms tumor, multiple myeloma, renal cancer, and cholangiocarcinoma [23,24,25]. Moreover, miR-19b overexpression has been found to inhibit the tumor suppressor PP2A by targeting the PP2A regulatory subunit PPP2R5E, thereby promoting tumor proliferation in non-small cell lung cancer (NSCLC) cells [26]. The role of miR-19b in CRC has been also studied in several works. During early colon cancer evolution, the expression of miR-19b was found confined to the epithelial cells, with an increased expression in the transitional zone from normal to adenomatous tissue [27]. MiR-19b has been identified in several studies to promote CRC cell proliferation [28,29], migration, and invasion [30]. In addition, a recent study has shown that the c-MYC-driven expression of miR-19b represses the proapoptotic protein Bcl2-like 11 (BIM) in CRC cells [31]. At the therapeutic level, miR-19b has been found to confer resistance to standard induction chemotherapy agents used in CRC treatment such as 5-fluorouracil (5-FU) and oxaliplatin. Jiang and colleagues experimentally confirmed that this miR mediates the resistance of CRC cells to oxaliplatin-based chemotherapy via SMAD Family Member 4 (SMAD4) [29]. Another work confirmed the role of miR-19b regulating sensitivity of CRC to oxaliplatin and demonstrated that the exosomal release of this miR is involved in the acquisition of an oxaliplatin-resistant phenotype [32]. Moreover, miR-19b has been proposed to play a role in the mechanism of 5-FU resistance in CRC cells and has been found to be overexpressed in 5-FU-resistant CRC cell line models [33]. Regarding the data available in the literature for this miR as a biomarker in CRC, circulating serum miR-19b has been proposed as a potential marker with diagnostic value for inflammatory bowel disease and colonic polyps [34]. Another study has shown that a signature of six miRs including miR-19b has diagnostic value for advanced adenoma and CRC detection [35]. Although the work by Cruz-Gil and co-workers has proposed that the expression of miR-19b would serve as a predictor of better prognosis in CRC patients [36], this work is the only discrepancy in the literature, and several other studies have highlighted its role as predictor of poor outcome. In fact, miR-19b overexpression has been proposed as an adverse prognostic marker for tumor recurrence and overall survival in CRC with liver metastases [37], and its clinical impact as a predictor of poor outcome in CRC has been further validated in several other studies [29,30]. In rectal cancer, there is only a study related to miR-19b. The DNA copy number (DCN) of the miR-17-92a-1 cluster host gene (MIR17HG), which includes miR-19b, has been analyzed by Molinari and colleagues in a cohort of 108 LARC patients. Although they did not find a significant association between MIR17HG DCN and response to neoadjuvant CRT, MIR17HG gene amplification was related to a lack of response, further suggesting the potential role of some miR included in this cluster in response to LARC neodjuvant treatment. In fact, the authors highlighted that the expression levels of the miRs included in this cluster should be evaluated in LARC patient cohorts [38]. Thus, the potential clinical impact of miR-19b in LARC remains to be investigated.

In our work, we evaluate for the first time the clinical significance of miR-19b as a predictor of both patient outcome and response to neoadjuvant CRT in LARC. We quantified the expression levels of this miR in a cohort that includes 121 LARC cases, observing that decreased miR-19b expression is a frequent event in this disease that significantly associates with different molecular and clinical parameters. Notably, we also observed that low miR-19b levels independently predicted better outcome in survival analyses as well as better pathological response to 5-FU-based preoperative CRT.

## 2. Experimental Section

### 2.1. Patients Tissue Samples

We selected retrospectively a total of 121 patients with LARC treated between 2007 and 2017 in University Hospital Fundación Jiménez Díaz (Madrid, Spain), and we studied the initial biopsies obtained by colonoscopy and prior to neoadjuvant CRT treatment. All patients were treated with neoadjuvant CRT and TME, and they were treated by the European guidelines recommendations with correct preoperative locoregional staging based on a magnetic resonance (MR), a transrectal ultrasound (TRUS), and a full body CT (computed tomography). The selection criteria were also adenocarcinoma, with operable disease, enough material, clinical follow-up data available, and no metastasis. TNM (tumor, node, metastases) staging was performed based on the 7^th^ American Joint Committee ion cancer (AJCC) staging system established for CRC. All patients gave written informed consent for tissue storage and analysis at the biobank of the Hospital Fundación Jiménez Díaz with the approval of the ethical committee with project number (2018/54).

### 2.2. Evaluation of Pathological Response

The tumor samples that resulted from the initial biopsies derived from colonoscopy were classified according to the CAP (College of American Pathologist) TNM, 7th ed. Two independent pathologists who were blinded to patient outcome evaluated tumor regression grade according to the modified Ryan classification that categorizes tumors into four levels of response: complete response, moderate response, minimal response, and poor response. A complete response score of 0 indicates no viable cancer cells; a moderate score of 1 indicates single cells or little groups of cancer cells; a minimal score of 2 indicates residual cancer outgrown by fibrosis; and a poor response score of 3 indicates minimal or no tumor kill with extensive residual cancer. According to clinical guidelines, every regression grade was compared with the primary tumor [39].

### 2.3. RNA Isolation

The RNA isolation from the tumor samples through FFPE (formalin-fixed paraffin-embedded) was performed following the protocol of the Recover All Total Nucleic Acid Isolation Kit Ambion (Thermo Fisher Scientific Waltham, MA, USA), and the RNA obtained was quantified with a Nanodrop Spectrophotometer (Thermo Scientific, Waltham, MA, USA). The FFPE samples were obtained from the paraffin block that allows avoiding contamination during storage for years.

### 2.4. Quantification of miRNA Expression Levels

A Recover all Total Nucleic Acid Isolation kit (Ambition) was used for the total RNA extraction following the manufacturer’s instructions. The reverse transcription of the samples was done with the TaqManHMicroRNA Reverse Transcription Kit (Applied Biosystems), and mature miRNAs were quantified by quantitative real-time reverse transcription polymerase chain reaction (RT-PCR) using TaqMan MicroRNA Assays (Applied Biosystems) specific for the miR-19b (reference number: 000396) and U6B (reference number: 001093) was used as an internal control. Reactions were carried out using an Applied Biosystems 7500 Sequence Detection System. Conditions: 95 °C for 10 min, followed by 45 cycles of 95 °C for 15 s and 60 °C for 1 min. Analysis of relative gene expression data was performed using the ΔCT method [40].

### 2.5. Statistical Analysis

SPSS Inc for windows was the software tool used for the statistical analyses. We employed the Chi-square test (Fisher exact test) based on bimodal distribution of data to analyze the correlation between miR-19b downregulation and the clinical and pathological variables. A cutoff for miR-19b expression was established using a receiver operating characteristic (ROC) curve as previously reported [18,19]. To assess the potential usefulness of mir-19b as a predictive biomarker, we choose the cutoff point that gave us the best sensitivity and specificity to discriminate rectal cancer pathologic response. Following this criteria, downregulation was considered when miR-19b expression levels [-ΔCT] were lower than 1.22. Event-free survival (EFS) was defined as the length of time from diagnosis of cancer until complications that the chemotherapy treatment was intended to prevent or delay (distal or local recurrence, last follow up or death. Overall survival (OS) was defined as the length of time from the date of pathological diagnosis to the date of the last follow-up or if the date of death. The K-M or Kaplan–Meier survival analyses were performed by means of log-rank test if the proportional hazard assumption was fulfilled and Breslow otherwise. The Cox proportional hazards model was adjusted, taking into consideration significant parameters in the univariate analysis. The guidelines followed to perform this article were the REMARK Guidelines (Reporting Recommendations for Tumor Marker Prognostic Studies) [41].

## 3. Results

### 3.1. Low miR-19b Expression Is a Common Alteration in LARC Patients that Associates with Molecular and Clinical PARAMETERS

We quantified the expression of miR-19b in a series of 121 LARC patients with clinical follow-up data available. From the cohort of 121 cases, 73 were males and 48 were females, with a median of age of 69 years (range: 36–86). Patient characteristics of the global cohort are shown in Table S1. We observed that the prevalence of low miR-19b expression was 38.8% (47 out of 121 cases) in our population. We next evaluated the potential association of low miR-19b downregulation with molecular and clinical parameters in our LARC patient cohort. Of note, low miR-19b levels correlated with lower tumor size after CRT (*p* = 0.003) and early pathological stage (*p* = 0.003). In addition, patients with low miR-19b expression tended to show lower tumor grade pre-CRT as well as lower lymph node positivity rates after CRT; however, statistical significance was not achieved in these cases (*p* = 0.103 and *p* =0.216, respectively). The associations between molecular and clinical parameters and miR-199b expression are shown in Table 1.

### 3.2. MiR-19b Is a Predictor of Pathological Response to Neoadjuvant CRT in Locally Advanced Rectal Cancer

**Figure 1:**
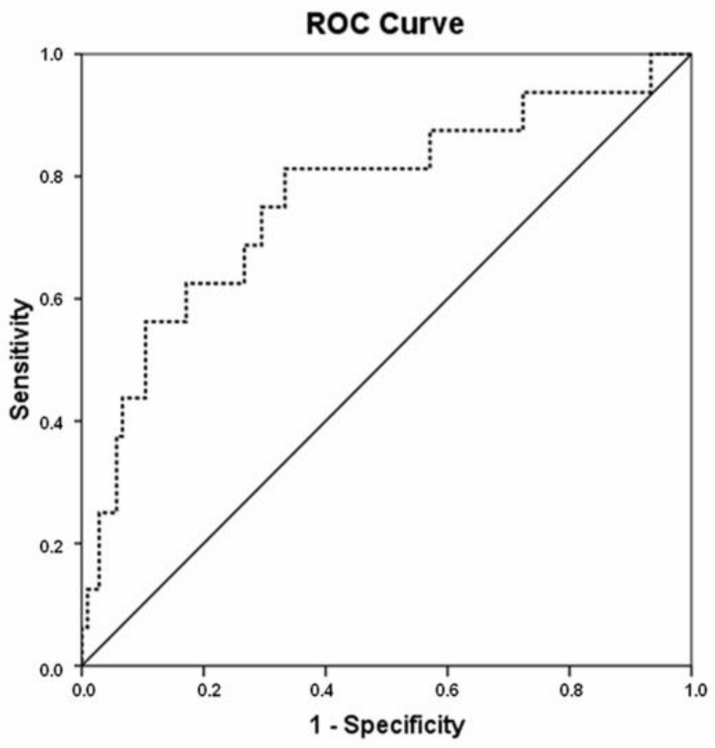
Receiver operating characteristic (ROC) curve to assess the usefulness of miR-19b to discriminate response to neoadjuvant chemoradiotherapy (CRT) in LARC. The dashed line is the coordinated point of the ROC curve. The solid line represents the reference diagonal line.

We next generated ROC curves in order to evaluate the potential clinical impact of miR-19b as a predictor of response to preoperative CRT in LARC. MiR-19b expression levels yielded an area under the curve (AUC) value of 0.765 (95% confidence interval (CI) = 0.626 to 0.905; *p* =0.001) with 81.3% specificity and 66.7% sensitivity in distinguishing LARC patient responders and non-responders to neoadjuvant CRT (Figure 1).

Thus, we observed that miR-19b expression was associated with response to neoadjuvant CRT (*p* < 0.001), and only 20.6% of those patients who did not show response had low miR-19b expression levels (Table 2).

In concordance with these data, we also observed a strong association between miR-19b expression and recurrence in our cohort, and miR-19b downregulation was found only in 11.5% of those cases that developed recurrence (Table 3).

Of importance, we performed multivariable logistic regression analyses including all clinical–pathological factors measured before the administration of neoadjuvant CRT. Interestingly, we observed that miR-19b expression levels quantified before neoadjuvant CRT serve as an independent predictor of pathologic response in LARC patients. The odds ratio for non-responders was 0.18 (95% CI = 0.06 to 0.57; *p* = 0.003) (Table 4).

### 3.3. MiR-19b Expression Determines Outcome in Locally Advanced Rectal Cancer Patients

**Figure 2:**
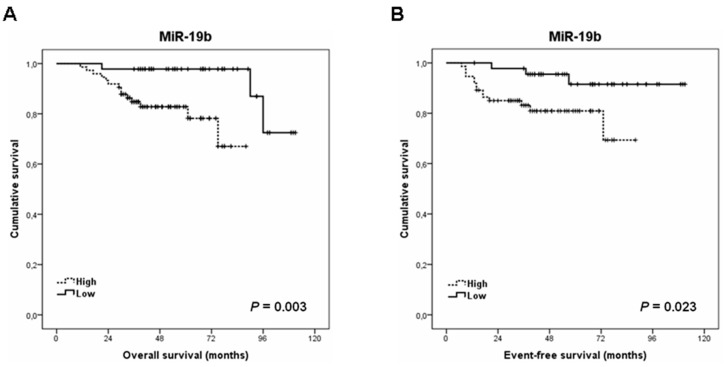
Clinical impact of miR-19b in LARC patient outcomes. Kaplan-Meier analyses for (**A**) overall and (**B**) event-free survival.

To evaluate the significance of miR-19b in LARC, we next investigated its potential clinical impact as a predictor of patient outcomes. For survival analyses, we included all the 121 LARC cases from our cohort, since clinical follow-up data were available for all them. Notably, we observed that low miR-19b expression defines a subgroup of patients that shows a markedly longer OS compared to those LARC cases with high miR-19b levels (105 versus 75 months, *p* = 0.003) (Figure 2A). Moreover, we also observed that low miR-19b expression determined significantly longer EFS in our patient cohort (105 versus 73 months, *p* = 0.023) (Figure 2B).

Furthermore, Cox proportional hazard regression analyses showed that pathological stage (hazard ratio (HR) = 3.484; 95% CI = 1.131 to 10.732; *p* = 0.030), lymph node positivity (HR = 3.747; 95% CI = 1.443 to 9.729; *p* = 0.007), and high miR-19b expression (HR = 0.085; 95% CI = 0.011 to 0.656; *p* = 0.018) were associated with poor outcome in the univariate analysis. Moreover, multivariate analyses revealed that miR-19b downregulation represents a favorable independent prognostic factor associated with longer OS in LARC patients treated with neoadjuvant CRT (HR = 0.093; 95% CI = 0.012 to 0.727; *p* = 0.024) (Table 5).

Notably, Cox proportional hazard regression analyses were also performed for event-free survival. Similar to OS, we found significance in univariate analyses for pathological stage (HR = 3.519; 95% CI = 1.145 to 10.814; *p* = 0.028), lymph node positivity (HR = 4.045; 95% CI = 1.55 to 10.522; *p* = 0.004), and high miR-19b expression (HR = 0.258; 95% CI = 0.073 to 0.910; *p* = 0.035). However, only miR-19b retained significance in multivariate analyses (HR = 0.268; 95% CI = 0.074 to 0.965; *p* = 0.044), indicating that it represents an independent predictor of EFS in our cohort of LARC patients (Table S2).

**Figure 3:**
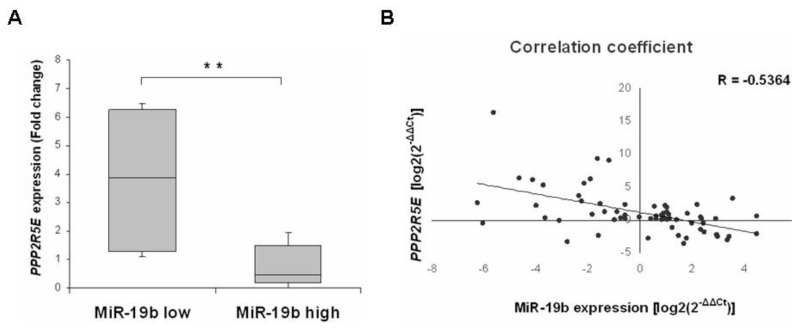
Evaluation of the miR-19b/PPP2R5E axis in LARC; (**A**) Box-plot showing *PPP2R5E* levels in LARC patients with low and high miR-19b expression; ** *p* < 0.001; (**B**) Scatter plot showing the negative correlation between miR-19b and *PPP2R5E* expression in 63 LARC patients.

Furthermore, we next analyzed the potential relevance of the miR-19b/PPP2R5E axis in LARC. Thus, *PPP2R5E* expression could be quantified in 63 LARC cases with enough material available from our patient cohort. Interestingly, we observed that the subgroup of patients with low miR-19b expression showed significantly higher *PPP2R5E* levels (*p* < 0.001) (Figure 3A), and we also found a negative correlation between miR-19b and *PPP2R5E* expression in our patient cohort (Figure 3B).

As expected, high *PPP2R5E* and low miR-19b expression were significantly associated in our series of LARC patients (*p* < 0.001) (Table S3), and PPP2R5E was also able to predict response to neoadjuvant CRT (*p* = 0.022) (Table S4).

## 4. Discussion

Nowadays, the clinical decisions in LARC are made based on clinical variables measured prior to neoadjuvant CRT. However, around 30% of cases do not show any response to preoperative CRT, and surgical resection should have been done from the beginning [35]. Although some potential biomarkers have been described in this context, none of them have been established and incorporated in the clinical routine [11,36]. Therefore, the identification of biomarkers predictive of response to neoadjuvant treatment still remains necessary and a challenge to optimize the clinical management of LARC patients. Here, we evaluate the potential clinical impact of miR-19b in this disease, and we hypothesized that miR-19b could serve as a good candidate to predict both outcome and response to neoadjuvant CRT in LARC patients based on several considerations. First, although its role in rectal cancer has not been investigated yet, this miR has been reported in several prior studies to predict outcome in CRC patient cohorts [29,30,36,37], suggesting that it could be a useful biomarker to predict outcome also in LARC. Second, miR-19b has been described to regulate the sensitivity of CRC cells to 5-FU [32], which could be directly related with the response to a neoadjuvant treatment based on this chemotherapy agent. Moreover, our group reported that PP2A inhibition is an alteration that confers 5-FU resistance in CRC and described the downregulation of PPP2R5E as a contributing mechanism to inactivate this phosphatase [42,43]. PPP2R5E is a PP2A regulatory subunit [44], and its deregulation has been reported to have important implications in both human cancer and cognitive disorders such as Alzheimer disease [26,45,46]. Interestingly, PPP2R5E has been identified as a direct target of miR-19b [26], further suggesting that this miR could be involved as a regulator of 5-FU sensitivity in LARC through PP2A regulation. Third, the work by Molinari and colleagues analyzed the DCN of MIR17HG, observing that MIR17HG gene amplification, which includes miR-19b, was related with a lack of response in LARC [38].

Our results showed that low miR-19b levels correlated with lower tumor size and early pathological stage, and that those cases with decreased miR-19b expression tended to show a lower lymph node positivity rate after-CRT (Table 1). In concordance with these findings, Zhang and colleagues described in their work increased miR-19b expression levels in the subgroup of CRC patients with lymph node metastasis compared with those patients with no lymph node metastasis. Moreover, these authors also found a higher miR-19b expression in the subgroup of patients with distal metastasis [30], and we observed here that high miR-19b levels are markedly associated with recurrence (Table 3). Furthermore, we found that those patients with low miR-19b expression had longer OS and EFS (Figure 2) and that this miR serves an independent predictor of prognosis in LARC (Table 5 and Table S2), and these findings would further supported by the fact that in previous works in the literature, high miR-19b has been associated with shorter survival in CRC patients [29,30,37]. The retrospective nature of the study and the lack of validation in larger independent cohorts are relevant limitations of our work that suggest taking the conclusions with caution. However, the present study is the first one to evaluate the clinical relevance of miR-19b in LARC. Finally, we show here that miR-19b levels have a marked predictive value of response to neoadjuvant CRT in our cohort of LARC patients, which suggests that this miR is probably regulating the sensitivity of the tumor cells to 5-FU. It would be very interesting to evaluate this issue in forthcoming studies as well as determine the molecular mechanism of action. In this way, it could be of interest to analyze the involvement and relevance of a potential miR-19b-mediated PP2A inhibition through the negative regulation of PPP2R5E. We found here a negative correlation between miR-19b and PPP2R5E expression (Figure 3), which would be suggesting the role of miR-19b as a negative regulator of PPP2R5E in LARC patients. The fact that PPP2R5E was also able to predict response to neoadjuvant CRT in our patient cohort (Table S4) highlights the potential significance of the miR-19b/PPP2R5E axis in regulating sensitivity to CRT, but this hypothesis needs to be confirmed in future functional studies. Of interest, PPP2R5E has been recently reported as a novel molecular target of allosteric PP2A activators with promising therapeutic implications in human cancer [46]. Moreover, the validation of our findings in an independent series of LARC patients is warranted to confirm the clinical impact of miR-19b and its usefulness as a robust biomarker in this disease. The lack of knowledge about a potential dependence of miR-19b of ethnic characteristics as well as the use of a unique cohort of 121 patients are relevant limitations that could lead to taking the conclusions of this study with caution before validation in an independent cohort. It would be also of relevance to evaluate its role as a biomarker in liquid biopsies and compare miR-19b expression between preoperative and postoperative samples from the same patient to strengthen its role in the progression of the disease.

## 5. Conclusions

In conclusion, our work shows evidence that miR-19b downregulation is a common event in LARC. Our results indicate that the subgroup of LARC patients with low levels of miR-19b independently predicts larger OS and EFS, and it could be used to anticipate good pathological response in this disease. However, it remains necessary to clarify the underlying mechanism of action of this miR to investigate its functional role and therapeutic value. Altogether, our findings highlight the potential usefulness of miR-19b as a predictive biomarker for pathological response and outcome in LARC patients treated with neoadjuvant chemoradiotherapy, which has to be fully validated in forthcoming studies including independent cohorts and liquid biopsies.

## Figures and Tables

**Table 1:** Association between clinical and molecular parameters and miR-19b expression levels in a cohort of 121 locally advanced colorectal cancer (LARC) patients.

	No. Cases	No. miR-19b Low (%)		No. miR-19b High (%)		*p*
MiR-19b	121	47 (38.8)		74 (61.2)		
Gender	121	47		74		0.605
Male	73	27	(37)	46	(63)	
Female	48	20	(41.7)	28	(58.3)	
Age	121	47		74		0.562
<60	40	17	(42.5)	23	(57.5)	
≥60	81	30	(37)	51	(63)	
ECOG ^1^	121	47		74		0.831
0	81	32	(39.5)	49	(60.5)	
1–2	40	15	(37.5)	25	(62.5)	
Clinical stage pre-CRT ^2^	121	47		74		0.285
II	9	5	(55.6)	4	(44.4)	
III	112	42	(37.5)	70	(62.5)	
Grade pre-CRT	112	42		70		0.103
Low	40	19	(47.5)	21	(52.5)	
Moderate-High	72	23	(31.9)	49	(68.1)	
ypT ^3^	121	47		74		0.003
0	16	13	(81.3)	3	(18.7)	
1	16	7	(43.8)	9	(56.2)	
2	38	12	(31.6)	26	(68.4)	
3	44	13	(29.6)	31	(70.4)	
4	4	0	(0)	4	(100)	
x	3	2	(66.7)	1	(33.3)	
ypN ^4^	121	47		74		0.216
N0	91	38	(41.8)	53	(58.2)	
N1	26	9	(34.6)	17	(65.4)	
N2	4	0	(0)	4	(100)	
Pathological stage	121	47		74		0.003
yp0	16	13	(81.3)	3	(18.7)	
ypI	43	15	(34.9)	28	(65.1)	
ypII	32	10	(31.3)	22	(68.7)	
ypIII	30	9	(30)	21	(70)	

^1^ ECOG = Eastern Cooperative Oncology Group; ^2^ CRT = Chemoradiotherapy; ^3^ ypT = tumor size after CRT; ^4^ ypN = pathological lymph node after CRT.

**Table 2:** Association between miR-19b expression levels and pathological response to neoadjuvant CRT in LARC patients.

Response to NCRT ^1^	No. Cases	Responders ^2^ (%)		Non-Responders ^3^ (%)		*p*
MiR-19b Expression	121	58		63		<0.001
Low	47	24	(72.3)	13	(27.7)	
High	74	34	(32.4)	50	(67.6)	

^1^ NCRT: neoadjuvant chemoradiotherapy; ^2^ Responders. Moderate or complete pathological response; ^3^ Non-Responders: poor or minimal pathological response.

**Table 3:** Association between patient relapse and miR-19b expression levels in LARC patients.

Recurrence	No. Cases	Yes (%)		No (%)		*p*
MiR-19b Expression	121	26		95		0.001
Low	47	3	(6.4)	44	(93.6)	
High	74	23	(31.1)	51	(68.9)	

**Table 4:** Univariate and multivariate logistic analyses for pathological response in the cohort of 121 LARC patients.

Response ^1^ vs. Non-Response ^2^		
	OR ^3^ (95% CI ^4^)	*p*
Gender, Male vs. Female	1.227 (0.558 to 2.98)	0.611
Age, < 60 vs. ≥60	0.778 (0.714 to 1.925)	0.587
Clinical stage pre-CRT ^5^, II vs. III	1.210 (0.709 to 2.064)	0.485
Grade pre CRT, Low vs. Moderate/High	1.021 (0.492 to 2.119)	0.956
ECOG ^6^, 0 vs. 1–2	1.174 (0.484 to 2.850)	0.722
miR-19b, High vs. Low	0.166 (0.071 to 0.390)	<0.001

^1^ Response: moderate or complete pathological response; ^2^ Non-response: poor or minimal pathological response; ^3^ OR: odds ratio; ^4^ CI: confidence interval; ^5^ CRT: chemoradiotherapy; ^6^ ECOG: Eastern Cooperative Oncology Group.

**Table 5:** Univariate and multivariate Cox analyses in the cohort of 121 LARC patients.

		Univariate OS ^1^ Analysis				Multivariate OS Cox Analysis			
		HR ^3^	95% CI ^2^		*p*	HR	95% CI		*p*
			Lower	Upper			Lower	Upper	
Gender					0.816				-
	Male	1.000							
	Female	0.888	0.326 to 2.418			-	-		
Age					0.225				-
	<60	1.000							
	≥60	2.167	0.621 to 7.564			-	-		
Pathological stage					0.030				0.490
	0-I	1.000				1.000			
	II-III	3.484	1.131 to 10.732			1.635	0.405 to 6.607		
ypT ^4^					0.139				-
	0–2	1.000							
	3–4	2.120	0.783 to 5.741			-	-		
ypN ^5^					0.007				0.105
	N-	1.000				1.000			
	N+	3.747	1.443 to 9.729			2.658	0.814 to 8.677		
ECOG ^6^					0.454				-
	0	1.000							
	1–2	1.450	0.548 to 3.836			-	-		
MiR-19b					0.018				0.024
	High	1.000				1.000			
	Low	0.085	0.011 to 0.656			0.093	0.012 to 0.727		

^1^ OS: overall survival; ^2^ CI: confidence interval; ^3^ HR: hazard ratio; ^4^ ypT: tumor size after chemoradiotherapy (CRT); ^5^ ypN: pathological lymph node after CRT; ^6^ ECOG: Eastern Cooperative Oncology Group.

## Data Availability

Data sharing is not applicable for this article.

## Supplementary Materials

The following are available online at https://www.mdpi.com/2072-6694/13/6/1456/s1, Table S1: Association between mir-19b and clinical and molecular parameters in 121 LARC patients, Table S2: Univariate and multivariate Cox analyses for EFS in the cohort of 121 LARC patients, Table S3: Association between *PPP2R5E* and miR-19b expression levels in LARC patients, Table S4: Association between *PPP2R5E* expression and pathological response to neoadjuvant CRT in LARC patients.
