# Supplementary material for: Low MicroRNA-19b Expression Shows a Promising Clinical Impact in Locally Advanced Rectal Cancer
Source: Cancers (Basel). 2021 Mar 22;13(6):1456. doi: 10.3390/cancers13061456 (PMC8005118; doi:10.3390/cancers13061456)
Supplement: Supplementary file 1 [file cancers-13-01456-s001.pdf]

cancers

MDPI

Low MicroRNA-19b Expression Shows a Promising Clinical Impact in Locally Advanced Rectal Cancer

Jaime Rubio, Ion Cristóbal, Andrea Santos, Cristina Caramés, Melani Luque, Marta Sanz-Alvarez, Sandra Zazo, Juan Madoz-Gúrpide, Federico Rojo and Jesús García-Foncillas

Table S1. Clinical and molecular characteristics of a series of 121 LARC patients.

		No. (%)	
**Sex**			
	Male	73	(60.3)
	Female	48	(39.7)
Age			
	<60	40	(33.1)
	≥60	81	(66.9)
ECOG[1]			
	0	81	(66.9)
	1-2	40	(33.1)
Site of primary tumor			
	Rectum	121	(100)
Clinical stage pre-CRT[2]			
	II	9	(7.4)
	III	112	(92.6)
Grade pre-CRT			
	Low	40	(35.7)
	Moderate-High	72	(64.3)
ypT[3]			
	0	16	(13.2)
	1	16	(13.2)
	2	38	(31.4)
	3	44	(36.4)
	4	4	(3.3)
	x	3	(2.5)
ypN[4]			
	0	91	(75.2)
	1	26	(21.5)
	2	4	(3.3)
Pathological stage			
	yp0	16	(13.2)
	ypI	43	(35.5)
	ypII	32	(26.5)
	ypIII	30	(24.8)
Neoadjuvant CRT			
	RT + 5-FU based	121	(100)
Recurrence			
	No	95	(78.5)
	Yes	26	(21.5)

[1]ECOG= Eastern Cooperative Oncology Group; [2]CRT= Chemoradiotherapy; [3]ypT= tumor size after CRT; [4]ypN= pathological lymph node after CRT.

Table S2. Univariate and multivariate analyses of factors predictive of event-free survival in the cohort of 121 LARC patients.

	**Univariate EFS[1] analysis**				**Multivariate EFS Cox analysis**			
	**HR[3]**	**95% CI[2]**		***p***	**HR**	**95% CI**		***p***
		Lower	Upper			Lower	Upper	
Gender				0.696				-
Male	1.000							
Female	0.820	0.303 to 2.219			-	-		
Age				0.201				-
<60	1.000							
≥60	2.255	0.648 to 7.851			-	-		
Pathological stage				**0.028**				0.492
0-I	1.000				1.000			
II-III	3.519	1.145 to 10.814			1.631	0.404 to 6.579		
ypT[4]				0.100				-
0-2	1.000							
3-4	2.306	0.852 to 6.243			-	-		
ypN[5]				**0.004**				0.066
N-	1.000				1.000			
N+	4.045	1.550 to 10.522			3.075	0.930 to 10.171		
ECOG[6]				0.349				-
0	1.000							
1-2	1.586	0.603 to 4.171			-	-		
MiR-19b				**0.035**				**0.044**
High	1.000				1.000			
Low	0.258	0.073 to 0.910			0.268	0.074 to 0.965		

[1]EFS: event-free survival; [2]CI: confidence interval; [3]HR: Hazard ratio; [4]ypT: tumor size after chemoradiotherapy (CRT); [5]ypN: pathological lymph node after CRT; [6]ECOG: Eastern Cooperative Oncology Group.

Table S3. Association between miR-19b and *PPP2R5E* expression levels in LARC patients.

	**No. Cases**	**MiR-19b low (%)**		**MiR-19b high (%)**		***p***
PPP2R5E	63	24		39		**<0.001**
Low	48	12	(50)	36	(92.3)	
High	15	12	(50)	3	(7.7)	

Table S4. Association between pathological response to neoadjuvant CRT and *PPP2R5E* expression in LARC patients.

**Response to neoadjuvant CRT[1]**						
Response	No. Cases	Responders[2] (%)		Non-Responders[3] (%)		*p*
PPP2R5E expression	63	26		37		**0.022**
Low	48	16	(33.3)	32	(66.7)	
High	15	10	(66.7)	5	(33.3)	

[1]CRT: chemoradiotherapy; [2]Responders: moderate or complete pathological response; [3]Non-Responders: poor or minimal pathological response.
